# From initial acceptance to reflective adjustment: psychological adjustment patterns among Taiwanese adolescents encountering misinformation

**DOI:** 10.3389/fpsyg.2026.1823402

**Published:** 2026-08-21

**Authors:** Kuo-Sung Lin, Ling-yu Zhang, Qingxiao Xie

**Affiliations:** Fujian College of Water Conservancy and Electric Power, Yong'an, China

**Keywords:** adolescents, misinformation, psychological adjustment, qualitative research, social cognitive theory

## Abstract

This qualitative study explores adolescents’ psychological adjustment when encountering online misinformation. Based on in-depth interviews with Taiwanese secondary school students, it examines how participants moved from initial acceptance of inaccurate information toward questioning, verification, recognition, and later adjustment. The analysis identified five interrelated patterns: initial acceptance and confusion, emerging doubt, verification, recognition and reflective reassessment, and subsequent adjustment. Although these patterns often appeared in a broadly temporal relationship, they did not constitute a fixed or universal sequence. Shaped by cognitive, emotional, and social-environmental factors, the patterns reflected adolescents’ developing self-efficacy and self-regulation. Findings suggest that self-efficacy beliefs, emotion regulation, and social and environmental feedback shaped participants’ movement among these patterns. Integrating Social Cognitive Theory with empirical evidence, the study advances a theory-informed account of media literacy development. Implications are discussed for educational interventions to enhance youth resilience to digital misinformation, including strengthening critical thinking and fact-checking skills and emphasizing the need for supportive environments. Study limitations and future research directions are also addressed.

## Introduction

Adolescents today grow up in an online environment where information circulates rapidly and unevenly, often blending verified knowledge with misinformation and fabricated content. During this formative period of cognitive and value development, young people engage actively with digital media while also facing heightened exposure to misleading information (Vraga and Bode, 2020). Empirical evidence suggests that many adolescents struggle to evaluate online sources, with studies indicating that a substantial proportion of secondary school students are unable to distinguish advertising from news content (Wineburg and McGrew, 2016). These difficulties point to persistent gaps in adolescents’ capacity to assess credibility in digital spaces (Wineburg and McGrew, 2016).

Adolescence is a distinctive developmental period for examining responses to misinformation. Executive functions, cognitive flexibility, and emotion-regulation capacities continue to develop across childhood and adolescence, while peer evaluation and social belonging become increasingly salient. These developmental characteristics do not imply that adolescents are uniformly incapable of evaluating information. Rather, they indicate that credibility judgments are made while reasoning strategies, regulatory capacities, and social identities are still being consolidated. Longitudinal evidence further indicates that emotion regulation and executive functioning remain developmentally interconnected during the school years (Halse et al., 2024), while young people’s everyday information practices are embedded in social relationships and identity-related concerns (Hassoun et al., 2023).

Challenges in evaluating online information carry psychological and social implications. Encounters with unverified rumors or sensational claims may evoke fear, anxiety, or confusion and can complicate young people’s trust judgments and social interactions. Recent research suggests that adolescents vary in their capacity to cope with online risks and recover from negative digital experiences, with digital resilience shaped by personal, relational, and environmental resources (Lahti et al., 2024; Qamaria et al., 2025).

Taiwan provides a particularly relevant context for examining these processes. Young people participate in a highly connected digital environment in which social media posts, interpersonal messaging, online news, influencer content, and politically or commercially contested claims circulate across platforms. A 2024 national survey reported that 95.05% of adult respondents in Taiwan had encountered misinformation, while 71.03% reported using fact-checking tools to verify suspicious information (Li, 2024). Although this survey did not focus specifically on adolescents, it illustrates the broader information environment in which Taiwanese young people develop credibility judgments through peer networks, family guidance, school education, platform use, and trust in institutional sources.

In response, educators and parents increasingly emphasize media literacy as a means of supporting adolescents’ critical engagement with online information. Educational systems in many countries have incorporated media literacy into formal curricula, with the aim of strengthening skills such as source evaluation, fact-checking, and reflective judgment (Gross and Balaban, 2025). While these initiatives address important competencies, they often rest on assumptions about how adolescents process misleading information. Less attention has been paid to the internal psychological processes through which adolescents move from initial confusion to more reflective understanding. Questions remain regarding how cognitive appraisal, emotional regulation, and behavioral adjustment unfold when young people encounter fake news.

Much of the existing literature relies on quantitative surveys to examine adolescents’ beliefs, sharing intentions, or levels of trust in online information. Although such approaches provide valuable patterns, they offer limited insight into how adolescents experience misinformation in everyday contexts or how social interactions shape their responses over time (Vraga and Bode, 2020). Qualitative studies remain comparatively scarce. Emerging work suggests that adolescents’ engagement with information is deeply embedded in peer relations and social environments, where personal judgment develops alongside social feedback and collective sense making (Hassoun et al., 2023).

To address this gap, the present study adopts a qualitative approach based on in-depth interviews with adolescents. By inviting participants to recount their encounters with fake news in their own terms, the study seeks to capture the lived processes through which initial acceptance, doubt, verification, recognition, and adjustment take shape. This approach allows for closer examination of how emotional reactions, cognitive reflection, and social mediation interact as adolescents navigate misinformation in their daily digital lives.

## Research objectives and questions

This study examines how adolescents adjust psychologically as they move from initial acceptance of misinformation toward recognition and correction of inaccurate beliefs. The focus lies on adolescents’ lived experiences as they encounter, interpret, and respond to misleading information in everyday digital contexts. The research is guided by the following questions.

First, how do adolescents describe their initial interpretations, feelings, and behavioral responses when encountering online information that they later recognize as inaccurate or misleading?

Second, what informational contradictions, interpersonal feedback, or personal reflections prompt adolescents to question an initially accepted claim, and how do they respond to the resulting uncertainty?

Third, what actions do adolescents take to assess the credibility of information, and what cognitive or emotional experiences do they report during verification?

Fourth, what emotional responses and reflective thoughts arise when adolescents come to realize that earlier beliefs were mistaken?

Finally, following recognition of misinformation, what changes do adolescents report in their source-evaluation habits, sharing decisions, trust judgments, and use of verification resources?

The analysis draws on Social Cognitive Theory to examine the reciprocal relationships among personal factors, behavior, and environmental influences in shaping adolescents’ responses to misinformation (Bandura, 1986, 2009). By tracing these interactions across different patterns of adjustment, the study aims to contribute to a more grounded understanding of adolescents’ media literacy development and psychological coping processes. The findings also offer practical insights for educators seeking to support adolescents’ reflective engagement with digital information in complex and socially embedded media settings.

## Literature review

### Social cognitive theory and media message processing

Social Cognitive Theory, proposed by Bandura (1986), emphasizes the reciprocal relations among personal factors, behavior, and environmental conditions. The theory suggests that individuals acquire knowledge not only through direct experience but also through observing others and interpreting the outcomes of their actions. Within the field of mass communication, Social Cognitive Theory has been applied to explain how media audiences form attitudes and behavioral responses (Bandura, 2009).

For adolescents who engage extensively with social media, observational learning plays an important role in shaping how information is understood and evaluated. Observing peers, influencers, or other visible figures discussing or sharing information contributes to adolescents’ judgments about credibility and relevance. This process reflects Bandura’s view that social modeling remains a central mechanism for learning in mediated environments.

Social Cognitive Theory further highlights the roles of self-efficacy and self-regulation. Self-efficacy refers to individuals’ beliefs about their capacity to perform actions required to achieve specific goals (Bandura, 1991). In the context of misinformation, adolescents with stronger self-efficacy tend to evaluate information more carefully, resist sharing unverified content, and attempt to correct inaccuracies. Recent studies indicate that confidence in one’s own evaluative abilities is closely associated with more cautious responses to misleading information (Paciello et al., 2023). In contrast, adolescents with lower self-efficacy may hesitate to verify information or may be more likely to conform to social pressure when sharing sensational content.

Self-regulation also shapes adolescents’ engagement with online information. This capacity involves managing cognitive and emotional responses during information processing. Effective self-regulation allows adolescents to moderate emotions such as fear or anger elicited by emotionally charged content, which supports more reflective judgment. Empirical findings suggest that stronger self-regulatory efficacy contributes to healthier cognitive and social development in online environments (Paciello et al., 2023). Taken together, Social Cognitive Theory provides a useful framework for understanding how adolescents navigate misinformation through the interaction of internal beliefs, observed social cues, and regulatory processes (Bandura, 1986, 2009; Corbelli et al., 2023; Paciello et al., 2023).

### Adolescents and misinformation: susceptibility and behavioral patterns

As misinformation circulates widely across digital platforms, scholars have increasingly examined how adolescents respond to misleading content. Due to developing digital literacy and heightened sensitivity to peer influence, adolescents are often identified as particularly vulnerable to misinformation (Breakstone et al., 2021; Notley and Dezuanni, 2019). Large-scale assessments of students’ online reasoning further show that many adolescents struggle to evaluate the credibility of digital sources and frequently rely on superficial cues such as layout or apparent authority (McGrew et al., 2018). Research conducted among Spanish adolescents shows that although many are aware of the existence of fake news, they tend to underestimate risks related to source credibility and rarely engage in systematic questioning of online information (Herrero-Diz et al., 2021).

Studies have also explored adolescents’ motivations for sharing misinformation. Herrero-Diz et al. (2020) identify several common reasons for sharing misleading content through instant messaging, including entertaining others, attracting attention, maintaining peer relationships, and warning others about perceived threats. These motivations reflect the social dimensions of information sharing, where relational concerns and self-expression often guide behavior (Herrero-Diz et al., 2020; Tandon et al., 2021).

The concept of Fear of Missing Out has been highlighted as another relevant factor. Beyens et al. (2016) note that adolescents who experience Fear of Missing Out are more likely to engage rapidly with trending online content. Concern about exclusion from peer conversations can prompt adolescents to share or follow information without verifying its accuracy, which contributes to the rapid spread of misinformation within youth networks (Beyens et al., 2016).

### Research gaps and positioning of this study

While existing research offers valuable insights into adolescents’ interactions with misinformation, much of the literature relies on cross-sectional survey designs that focus on perceptions, trust levels, or self-reported behavioral intentions. Such approaches provide limited understanding of how adolescents experience misinformation as a process that unfolds over time. In particular, fewer studies examine how adolescents move from initial exposure to later recognition, reflection, and adjustment.

In addition, a large proportion of prior research has been conducted in Western contexts. Given Social Cognitive Theory’s emphasis on environmental influences, adolescents in Asian settings may display different patterns of information processing shaped by family structures, educational norms, authority relations, and collectivist values. These contextual dimensions remain underexplored in existing studies.

This study addresses these gaps through in-depth qualitative interviews that capture adolescents’ lived experiences with misinformation. While quantitative research identifies how frequently certain behaviors occur, qualitative methods allow closer examination of how adolescents describe their emotional reactions, cognitive struggles, and social interactions. By reconstructing these psychological trajectories through participants’ narratives, this study interprets the interplay between individual and environmental factors through Social Cognitive Theory. This approach contributes to a more nuanced understanding of adolescent media literacy and offers insights relevant for educational practice (Hassoun et al., 2023).

### Theoretical framework: social cognitive theory

Social Cognitive Theory was adopted as the theoretical framework for examining the reciprocal relationships among personal factors, behavior, and environmental influences in adolescents’ responses to misinformation. The theory proposes that individuals’ cognition, emotion, and beliefs interact with their behavior and social environments rather than operating independently. In the present study, this perspective was used to examine how adolescents’ self-efficacy beliefs, emotional responses, information-checking behavior, and interactions with peers, family members, teachers, and media sources shaped their responses to questionable information.

Self-efficacy was particularly relevant to the present study because adolescents differed in their confidence in evaluating information, searching for evidence, and resisting the impulse to share unverified content. Emotion regulation was also considered because emotionally charged information could influence whether participants reacted immediately or paused to reconsider a claim. Environmental influences included feedback from peers, guidance from family members or teachers, and credibility cues associated with media platforms and information sources.

Social Cognitive Theory did not prescribe a predetermined sequence of adjustment stages in this study. Rather, it served as a theoretical lens for interpreting the relationships among participants’ beliefs, emotions, actions, and social contexts. The five patterns presented in the findings were developed through analysis of the interview data and were subsequently interpreted in relation to Social Cognitive Theory.

## Methodology

This study adopts a qualitative research design to examine adolescents’ psychological adjustment when encountering misinformation. In-depth interviews were used as the primary method of data collection, followed by thematic analysis to interpret participants’ accounts. This approach allows close attention to adolescents’ lived experiences and to the cognitive, emotional, and social processes involved in responding to misleading information (Braun and Clarke, 2021). The following sections describe participant recruitment, data collection procedures, analytic strategies, and steps taken to ensure research trustworthiness.

## Participants and sampling

Participants consisted of ten adolescents aged between thirteen and eighteen years, including five males and five females, all enrolled in either junior high or senior high school. Purposive sampling was used to recruit participants with relevant experience of encountering misinformation online (Palinkas et al., 2015; Tracy, 2024). Initial participants were introduced through schoolteachers and youth group leaders who were familiar with students interested in digital media issues. These participants then referred peers through snowball sampling, which helped broaden the range of experiences represented in the study (Braun and Clarke, 2021).

Recruitment included adolescents from both urban and suburban schools in order to capture variation in everyday media environments. Prior to participation, informed consent was obtained from both adolescents and their guardians after clear explanation of the study’s purpose, procedures, and intended academic use of the data (Alderson and Morrow, 2020). To protect privacy, all participants were assigned pseudonyms or identification codes such as S1 or S2, and any potentially identifying information was removed from transcripts.

Although the sample included ten participants, data collection followed the principle of saturation. By the eighth and ninth interviews, participants’ descriptions increasingly elaborated the developing patterns of adjustment, while few substantially new aspects of the phenomenon were identified (Braun and Clarke, 2021). Given the depth of the interviews and the focused research aim, the sample was considered adequate for an exploratory, context-bound analysis.

## Data collection procedures

Data were collected through individual semi-structured interviews conducted on a one-to-one basis (Tracy, 2024). Each interview lasted approximately thirty to forty-five minutes and took place in a quiet and familiar setting such as a school meeting room or library study space. All interviews were conducted by the principal investigator or trained research assistants and were audio-recorded with participants’ permission.

The interview guide was designed to align with the research objectives and included open-ended questions covering six thematic areas. Participants were first invited to recall a recent experience in which they believed an online message or news item that was later shown to be false. They described where they encountered the information and their initial reactions. They were then asked why the content appeared credible, focusing on features of the message, source, or sender, as well as the emotions they experienced at that time.

Subsequent questions explored the emergence of doubt, including what triggered questioning and what forms of internal conflict arose. Participants were also asked about actions taken to verify the information, such as online searching, consulting fact checking resources, or seeking advice from teachers, parents, or peers. Attention was given to difficulties encountered during this process, including uncertainty about where to begin or feeling overwhelmed by information.

Participants then described how they eventually realized the information was inaccurate and reflected on their emotional responses at that moment. The interview concluded with questions about post-experience adjustment, focusing on whether participants reported changes in attitudes or behaviors toward online information and how they interpreted the experience in terms of personal learning or growth.

Follow-up prompts were used to encourage elaboration and clarification, particularly regarding emotional and cognitive experiences. Interviews were conducted in a neutral and nonjudgmental manner to support open narration (Braun and Clarke, 2021). All recordings were transcribed verbatim and checked for accuracy, resulting in approximately fifty pages of text for analysis.

### Data analysis and interpretation

Interview data were analyzed using thematic analysis, drawing on the foundational framework proposed by Braun and Clarke (2006) and its later refinement as reflexive thematic analysis (Braun and Clarke, 2021). Analysis began with repeated reading of all transcripts to achieve familiarity with the data and to note preliminary observations. Researchers then conducted line by line coding independently, assigning brief labels to segments relevant to the research questions. For example, statements reflecting uncritical acceptance of information were coded separately from those describing moments of doubt.

The research team then met to discuss and refine initial codes. Related codes were grouped into broader categories, with particular attention to sequences that reflected change over time. Through iterative discussion and comparison with the original transcripts, candidate themes were reviewed and adjusted to ensure they accurately represented participants’ intended meanings.

The developing themes were subsequently interpreted through Social Cognitive Theory, with particular attention to self-efficacy beliefs, emotion regulation, behavior, and environmental influences (Bandura, 1986). This theoretical lens supported interpretation of how personal factors, behavior, and social contexts interacted across the different patterns of adjustment. Ultimately, five main themes were developed and organized as interrelated patterns that frequently displayed temporal relationships without constituting a fixed chronological sequence.

To enhance credibility, member checking was conducted after preliminary analysis (Braun and Clarke, 2021; Tracy, 2024). Six participants reviewed a written summary of the findings and confirmed that the interpretations reflected their experiences. Minor feedback was incorporated to better align descriptions with adolescent language and perspectives. Triangulation was also applied by reviewing selected social media posts or comments shared voluntarily by participants, which supported contextual understanding but were not coded directly. Together, these procedures contributed to the credibility and confirmability of the analysis.

## Positionality statement

The interviewing team consisted of the first author and two trained non-author research assistants, while the first and third authors conducted the coding and thematic analysis. The first author has backgrounds in information management, communication, qualitative research, and youth work. One non-author research assistant has backgrounds in education and psychology, while the other had training in semi-structured interviewing and experience using thematic analysis and NVivo. The third author has a background in digital media research and experience using thematic analysis and NVivo.

None of the interviewers or coders had any prior teaching, supervisory, evaluative, personal, or other pre-existing relationship with the participants. The research team was familiar with Taiwanese secondary-school education, adolescents’ social media use, youth-work settings, and Taiwan’s broader digital-media environment.

The authors acknowledge that their disciplinary backgrounds and prior research interests in media literacy, misinformation, Social Cognitive Theory, and adolescents’ digital-media behavior could have influenced data collection and interpretation. To address this potential influence, the interviewers used open-ended questions and maintained a neutral and nonjudgmental tone. The two coders analyzed the transcripts independently before discussing differences in coding and theme interpretation. The research team repeatedly returned to the original transcripts, retained accounts that did not fit a straightforward progression, and conducted member checking with six participants. These procedures supported reflexive awareness and enhanced the transparency and trustworthiness of the analysis.

## Research findings

**Figure 1:**
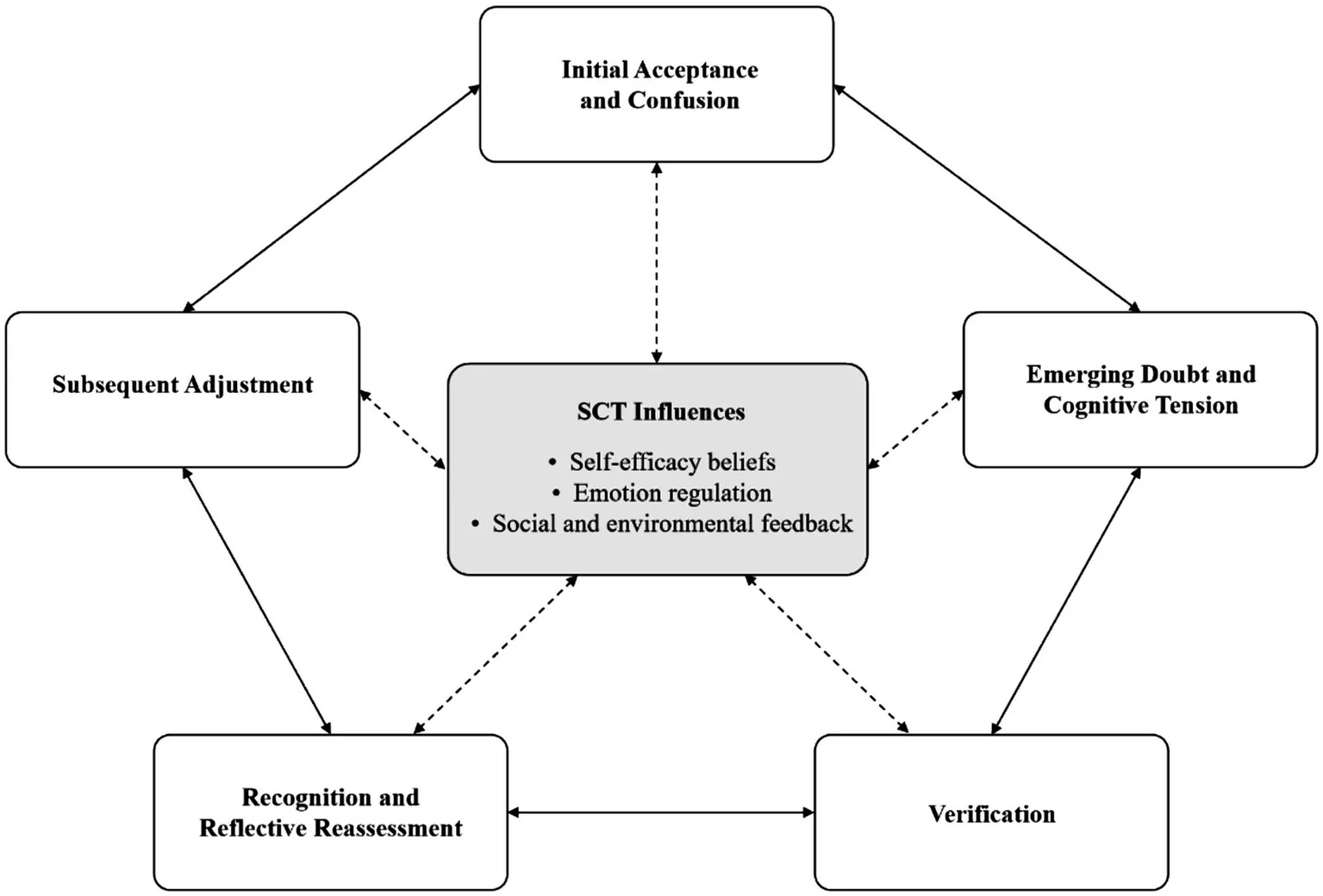
Interrelated patterns in adolescents’ psychological adjustment to misinformation. The figure represents recurring and potentially recursive relationships identified in participants’ accounts rather than a fixed or universal stage sequence. Bidirectional arrows indicate that participants could move between adjacent patterns, including repeated movement between doubt and verification, while Social Cognitive Theory influences interact with all five patterns.

The analysis developed five interrelated patterns in participants’ accounts: initial acceptance and confusion, emerging doubt, verification, recognition and reflective reassessment, and subsequent adjustment. These patterns frequently appeared in a broadly temporal relationship, but they should not be interpreted as fixed or universal developmental stages. Some participants moved repeatedly between doubt and verification, and the form of later adjustment varied across individual accounts. Each pattern includes several subthemes, summarized in Table 1. The sections below describe each pattern and draw on adolescents’ words to clarify how these experiences were lived and interpreted. Figure 1 summarizes these relationships.

**Table 1:** Psychological adjustment process analysis

Pattern	Emotional traits	Cognitive/behavioral reactions	Summary analysis	Mediating factors
Initial Acceptance and confusion	Fear, panic, blind trust	Immediate belief and sharing, lack of evaluation	Adolescents are vulnerable to emotional and source-based cues	Source credibility, emotional triggers
Emerging doubt and cognitive tension	Embarrassment, self-doubt, conflict	Emerging suspicion, cognitive dissonance	External cues trigger transition into reflective doubt	Peer reminders, contradicting info, internal conflict
Verification	Anxiety, confusion, information overload	Active verification, cross-checking, consulting others	Verification indicates activation of self-efficacy and digital agency	Media literacy, self-efficacy, accessibility of resources
Recognition and reflective reassessment	Shame, regret, anger	Reflective thinking, emotional confrontation	Emotional and reflective processes lead to stronger awareness	Emotion regulation, reflective thinking, value shifts
Subsequent Adjustment	Calmness, confidence, responsibility	Established habits, improved skepticism and resilience	Behavior stabilizes, forming resilient media habits	Digital resilience, critical adaptation, independent judgment

### Initial acceptance and confusion

The initial acceptance and confusion pattern refers to adolescents’ first encounter with misinformation. Many participants described an initial period of uncritical acceptance and uncertainty. Because misinformation often resembles legitimate news and is presented in attention-grabbing formats, participants reported being persuaded by surface cues that appeared credible. They also noted that trust was higher when the information came from familiar sources, or when the content matched existing beliefs and emotional concerns.

Emotional reactions were often immediate and strong. Participants described shock, excitement, or fear, and several accounts suggested that these feelings strengthened belief in the message. A sixteen-year-old participant described her response to a food safety rumor:

“When I saw the message, I was honestly terrified. It said a popular snack brand contains carcinogens. The more I read, the more scared I became. I immediately believed it and threw away all the snacks at home.” (S4).

A seventeen-year-old participant described how a celebrity death rumor affected him:

“I was shocked. I did not question it at all. I was sad for half the day and even shared it with a friend.” (S6).

These narratives suggest that emotionally charged content can narrow attention and reduce questioning at the moment of exposure. Participants also highlighted the role of source cues. Many said they were more likely to accept misinformation shared by a close friend, family member, or influencer. This aligns with Social Cognitive Theory, which emphasizes that individuals often use others’ behavior and attitudes as reference points for judgment (Bandura, 1986). Several participants explained their reasoning in simple terms: if a trusted person shared it sincerely, it felt safer to believe.

Presentation cues also shaped early belief. Messages formatted like news alerts, or those using charts, photos, or supposed expert quotes, were often treated as reliable. At this stage, participants rarely described active motivation to check accuracy. The sense that many people were discussing the same message also supported belief. Overall, this pattern reflects an early period in which critical evaluation is muted. Participants described passive acceptance combined with strong emotional engagement, which set the conditions for later shifts.

### Emerging doubt and cognitive tension

The emerging doubt pattern began when participants started questioning information they had previously accepted. Doubt was usually triggered by one of three types of cues.

First, some participants encountered contradictory information from sources they viewed as credible, such as teachers or established news outlets. One participant described believing a rumor for several days until a teacher provided context, which made the story feel questionable.

Second, doubt was sometimes prompted by direct challenge from others. A fifteen-year-old participant described being corrected by a classmate after sharing a rumor:

“I forwarded the news in our class group chat, and one of my classmates immediately messaged me saying it was fake, asking why I’d believe something like that. I froze. That’s when I started to doubt myself.” (S9).

Third, some participants described personal reflection after emotional intensity subsided. A seventeen-year-old participant explained:

“That article made me so anxious at first. But two days later, after calming down, I noticed a lot of shocking claims with no evidence. That’s when I started thinking I might’ve been fooled.” (S7).

During this stage, many participants described tension between competing concerns. They suspected the message might be wrong, but they also resisted admitting this, especially if they had already shared it. An eighteen-year-old participant expressed this conflict clearly:

“I was kind of doubting it… but I did not want to believe I’d been tricked. Part of me hoped it was true so I would not be wrong. But at the same time, something just felt off. It was really conflicting.” (S3).

Some participants tried to suppress doubt and looked for confirming information to protect their initial belief. Others accepted uncertainty and moved toward verification. Social Cognitive Theory helps explain these differences through personal factors such as self-efficacy and confidence in judgment (Bandura, 1991). Participants who described higher confidence in their evaluative skills were more willing to confront uncertainty. Others described avoidance, which can be understood as a response to discomfort and the desire to protect self-image, consistent with work on confirmation bias (Nickerson, 1998). Environmental factors also mattered. Several participants said that when peers around them believed the message, they hesitated to express doubt because they feared being singled out. This pattern therefore involved both cognitive questioning and social pressure and became a key turning point in participants’ accounts.

### Verification: actions to check credibility

The verification pattern described the period when participants took steps to assess accuracy after doubt took hold. Participants used several strategies.

The most common action was online search and cross-source comparison. Many participants described searching keywords and checking whether mainstream news outlets reported similar information. One participant described noticing that only unreliable blogs repeated the story, which led him to seek debunking material:

“I had a weird feeling, so I Googled the keywords. I noticed that none of the major news websites reported on it. Only some weird blogs were talking about it. Then I found a blog post that explained it was fake news, and that’s when I knew the message I first saw wasn’t true.” (S7).

This kind of comparison reflects lateral reading practices discussed by Wineburg and McGrew (2019), where credibility is assessed by leaving the original source and checking how information is handled elsewhere. Only a minority of participants described this strategy in a deliberate way, but those who did often found it effective.

Another common strategy was consulting trusted individuals. Participants frequently turned to teachers or family members, especially when the topic involved health or safety. One participant brought a questionable article to a teacher:

“Our biology teacher often talks about food safety, so I showed him the article. He read it and immediately said it was fake and explained why it could not be true.” (S5).

A fourteen-year-old participant described seeking guidance from her father, a doctor:

“I read online that a certain cold medicine had toxic side effects. I was too scared to take it, so I asked my dad. He’s a doctor. He said it was a rumor, and I felt much better.” (S10).

A smaller group of participants used fact-checking platforms. Some mentioned Taiwan’s Rum Toast project or international sites such as FactCheck.org. Those who used such tools often described higher confidence in their ability to verify claims, which aligns with Bandura’s view that self-efficacy supports proactive use of resources (Bandura, 2009).

Verification was not always straightforward. Participants described information overload and conflicting reports as common obstacles. A seventeen-year-old participant explained:

“I searched online and got tons of results. Some said it was true, others said it was fake. I read a few articles, but they all said different things, and I did not know what to believe anymore.” (S6).

Others described difficulty judging sources when posts included technical terms or statistics. One participant described discovering that a supposed debunking source was itself unreliable:

“I found a post that claimed to be the truth, but a few days later I learned even that so-called fact-check was fake. I was stunned. I did not know what to believe anymore.” (S2).

These accounts show that verification can involve repeated cycles of uncertainty. Even so, most participants reported that sustained effort eventually produced a clearer view of what was credible, which prepared the ground for recognition and reflective reassessment.

### Recognition and reflective reassessment

The recognition and reflective reassessment pattern referred to the point at which participants confirmed that information they had believed was false. Participants often described this as a sudden recognition, followed by strong emotion and reflection. Emotional responses varied. Some participants described shame or regret and linked these feelings to self-blame, especially if they had shared the misinformation. One participant said:

“When I found out it was fake, I felt super annoyed with myself. Like, how could I be so dumb to believe that?” (S9).

Anger was also common, often directed toward those who created or spread the misinformation:

“Thinking about how someone intentionally spread fake news and made me worry for nothing, yeah, I was mad!” (S4).

For some, this anger expanded into distrust of the broader information environment. An eighteen-year-old participant said:

“How much fake stuff is even out there online now? Feels like I cannot trust anything anymore. I’m becoming a bit cynical.” (S3).

Others described relief, especially when misinformation had caused anxiety:

“When I found out it was fake, I actually felt relieved. At least the world wasn’t as bad as I thought. I just worried for nothing.” (S10).

After the first emotional response, participants often described reflection on why they had believed the message, as they began to regulate intense emotions and regain cognitive composure, a process central to emotional self-regulation in children and adolescents (Lazarus and Costa, 2020). Their reflections tended to fall into three areas. First, some spoke about personal limits in judgment and the need to improve media literacy:

“Looking back, if I had checked more at the beginning, I would not have believed it. It just shows I trust the internet too easily. I really need to work on that.” (S6).

Second, some criticized the online environment and expressed stronger skepticism toward sources:

“What’s with these people spreading fake stuff? There are just so many rumors online. I seriously need to be more careful from now on.” (S5).

Third, some described changes in values and media habits, such as shifting attention toward more reliable information sources. One participant explained:

“I used to think Weibo was the fastest and coolest way to get news, but now I feel like official channels are more reliable. From now on, I’ll pay more attention to legit news and not believe all those unverified rumors.” (S1).

Recognition and reflective reassessment therefore combined emotional response with reconsideration of earlier judgments. It marked a turning point where participants acknowledged error and began to form a different stance toward online information.

## Subsequent adjustment

The subsequent adjustment pattern described how participants translated these experiences into more stable changes in practice. Many reported behavioral changes, especially reduced impulse to forward content. A participant said:

“Now, when I see something sensational, I will not forward it immediately. I’ll check it first and then decide whether to believe it.” (S4).

Others described adopting new habits such as subscribing to fact-checking accounts:

“I subscribed to some fact-checking accounts. When I see suspicious news, I’ll check if anyone has debunked it.” (S7).

Some participants also described acting as informal gatekeepers in peer networks:

“I got tricked once, so now when I see others spreading fake news, I step in and tell them it’s false and send them the source.” (S5).

Participants also described psychological shifts. Many reported greater vigilance and routine questioning of exaggerated claims. An eighteen-year-old participant described this in a metaphor:

“It’s like I have a mental question mark filter now. Whenever I see exaggerated headlines, I ask: Is this true? Where’s the evidence?” (S3).

Several participants also expressed greater confidence in dealing with misinformation after the experience, suggesting growth in self-efficacy. At the same time, a small number described stronger distrust that extended even to official sources:

“There’s fake news everywhere. I do not even trust official media anymore.” (S6).

These accounts suggest that adjustment can take different directions. Most participants described balanced caution, but a few moved toward broad skepticism. Participants also described strategies for coping with the information environment. Some curated their feeds by unfollowing unreliable accounts and following more credible sources:

“I unfollowed a few Weibo accounts that always post unreliable stuff and followed more credible news channels instead.” (S1).

Others delayed judgment and waited for additional information:

“Now when I see shocking news, I wait a day or two to see if more info comes out before deciding what to think.” (S8).

A small group took part in media literacy activities, including sharing personal experience to warn others:

“I wrote an article about my experience and submitted it to the school magazine to remind others to be careful about online rumors.” (S9).

Overall, this pattern reflects how adolescents incorporated experience into everyday practice. Participants described more cautious habits, stronger attention to credibility, and, in some cases, willingness to support peers. Taken together, the five interrelated patterns capture movement from early acceptance toward more deliberate judgment and practical strategies for coping with misinformation, without implying a fixed or universal sequence.

## Discussion

This study examined how adolescents describe their psychological adjustment after encountering misinformation. Based on in-depth interviews, we identified five interrelated patterns: initial acceptance and confusion, emerging doubt, verification, recognition and reflective reassessment, and subsequent adjustment. The discussion considers how this process can be understood through Social Cognitive Theory and how it connects with previous research on media literacy, emotion, and youth information practices. It also outlines implications for education and support in schools and families.

### Positioning of the study and analytic focus

This research was designed as an exploratory qualitative study that attends closely to adolescents’ accounts of how belief, doubt, and later reflection unfold in everyday digital contexts. The aim is not to claim representativeness for all adolescents. Instead, the analysis draws on detailed narratives to clarify psychological mechanisms that are often difficult to see in survey designs (Tracy, 2024). Social Cognitive Theory provides a useful framework for this purpose because it links personal factors, behavior, and environmental influences in a single explanatory model. The interviews show how agency, emotion regulation, and social cues interact across time, and they offer a grounded basis for later confirmatory work.

The contribution of the present study lies not in proposing a universal stage model, but in connecting psychological and behavioral responses that previous studies have often examined separately. Participants’ accounts linked initial emotional acceptance, emerging doubt, verification attempts, recognition of error, and later behavioral adjustment. At the same time, the patterns were not uniformly progressive: doubt did not always lead immediately to verification, verification sometimes produced further uncertainty, and later adjustment varied across participants. This process-oriented account therefore extends research that has primarily focused on misinformation belief, sharing intentions, source evaluation, or intervention outcomes as separate phenomena.

### Social cognitive theory and adolescent media literacy

The findings support the relevance of Social Cognitive Theory in the context of youth engagement with misinformation (Bandura, 1986). Across the five patterns, adolescents described reciprocal relations among personal factors, environmental conditions, and behavioral responses. Self-efficacy was particularly visible (Paciello et al., 2023). It shaped whether adolescents moved from doubt into verification and whether they regained confidence after recognising error. Paciello et al. (2023) distinguish active efficacy, such as taking steps to fact check, from inhibitory efficacy, such as resisting the impulse to share. Participants who described sustained verification and later adjustment often showed stronger active efficacy, while those who remained in confusion or hesitated at doubt described limited confidence or limited skills.

Environmental influences were also central. Peer circulation increased perceived credibility at the point of first exposure, while later guidance from teachers and parents helped participants evaluate claims and recover a sense of orientation. This pattern is consistent with Social Learning Theory, which highlights modelling and social support in learning processes (Bandura, 2009). Adolescents did not process information alone. They learned by watching how others reacted, by seeking confirmation, and by observing what was treated as trustworthy.

Behavioral change in the subsequent adjustment pattern also aligns with Social Cognitive Theory. Participants described shifts in media habits after being misled, including more cautious sharing, checking sources, and in some cases intervening in peer conversations. These changes reflect learning through experience and gradual strengthening of self-regulation (Jeong et al., 2012; Paciello et al., 2023). Taken together, the findings show a self-learning cycle in which adolescents relied on observation, managed emotional reactions, and revised practices based on reflection and social feedback.

### Links with previous research

The initial acceptance and confusion pattern aligns with research suggesting that adolescents often accept content shared by acquaintances with limited vigilance. It also supports experimental evidence that reliance on emotion is associated with greater belief in false news (Martel et al., 2020). Participants did not describe belief as purely cognitive. Their accounts show how fear, excitement, and shock can shape early acceptance and delay later questioning.

The study also echoes research on social motivations for sharing. Participants described sharing to remain socially connected, to entertain others, or to avoid being left out of peer discussions. These accounts align with research on Fear of Missing Out and youth sharing practices (Herrero-Diz et al., 2020; Tandon et al., 2021). At the same time, the interviews add detail about motivations for not sharing and for correcting misinformation. Several participants described shifting from being a sender to being a cautious peer who slows circulation. This role shift has received less attention in previous work and deserves further study.

Within the verification pattern, only a minority of participants described strategies consistent with lateral reading and the use of authoritative sources (Notley and Dezuanni, 2019; Wineburg and McGrew, 2019). Many relied on simple searching and reported difficulty assessing credibility, which is consistent with evidence that adolescents often struggle to evaluate online information (Wineburg and McGrew, 2016). The interviews highlight how this weakness shapes later emotions and reflection, not only accuracy outcomes. It becomes part of how adolescents interpret themselves and their environment.

A further contribution lies in the process perspective. Prior studies have often examined belief, sharing motives, or intervention effects as separate topics. This study links these elements by tracing a movement from initial trust through later doubt, verification efforts, and longer term adjustment. The pattern also resembles the experiential learning cycle described by Kolb (2014), in which concrete experience is followed by reflection, conceptual understanding, and later experimentation in practice. Participants described a similar movement as they learned from being misled and modified their media routines.

### Practical implications for education and support

The findings point to several practical considerations for schools and families.

First, adolescents often lacked stable verification habits. Media literacy education can include routine practice in cross-checking sources, judging website credibility, and using fact-checking resources (Notley and Dezuanni, 2019). Intervention research has shown that explicit instruction in evaluating sources and practicing civic online reasoning can significantly improve students’ ability to assess online information (McGrew, 2020; Pennycook et al., 2020). Case based exercises can help students learn how misinformation is constructed and how verification can proceed step by step.

Second, emotional response played a strong role in early belief. Educational programs can address how emotions shape judgment, especially when content triggers fear or anger. Instruction that supports emotional awareness and self-regulation can help adolescents slow down and evaluate before sharing or acting.

Third, peer influence shaped both belief and correction. Schools can support peer-based learning by encouraging students with interest in media literacy to lead activities that model careful sharing. Peer initiatives can also create norms that make it easier to express doubt without social penalty.

Finally, adolescents need a supportive environment for questioning. Schools and families can help by making trusted resources accessible and by treating doubt as a responsible response. Teachers can provide curated examples of reliable sources and can invite students to raise questions openly. At home, parents can respond with patience and guidance when adolescents ask about uncertain claims. When adolescents sense that inquiry is welcomed, they are more likely to verify carefully and less likely to rely on quick social cues.

## Limitations and future research directions

This study has several limitations that should be considered when interpreting the findings. First, the participants were drawn from a specific cultural and regional context in Taiwan. Cultural norms related to authority, family relations, and peer interaction may shape how adolescents respond to misinformation and how they seek guidance or verification. Caution is therefore required when extending these findings to adolescents in other societies. Future research could apply similar qualitative approaches in different cultural settings to examine whether the adjustment process follows comparable patterns or reflects context-specific variations.

Second, participation was voluntary and focused on adolescents who were willing to discuss their experiences with misinformation. This may have resulted in a sample that was relatively engaged or reflective about media use. Adolescents who rarely question online information or who remain unaware of misinformation may be less visible in this dataset. Future studies could seek broader recruitment strategies, including adolescents who report repeated exposure to misleading information without reflection, or those who are embedded in online communities where misinformation circulates more intensively.

Third, the study relied on retrospective accounts. Participants described experiences after they had already processed and reflected on them, which may introduce memory-related distortions. Emotional intensity, time lapse, or later reinterpretation could influence how events were recalled. Research designs that follow adolescents over time or examine encounters with misinformation as they occur could provide a more detailed view of how adjustment unfolds and how responses change across repeated exposures.

Future research may also benefit from closer attention to individual differences and content characteristics. In the present study, some participants described emotional reactions more openly than others, which may be related to gender norms or personal communication styles. Developmental differences may also matter as younger adolescents may have fewer cognitive resources for abstract evaluation than older youth. In addition, different types of misinformation may elicit distinct reactions. Health related rumors, political claims, and celebrity gossip may involve different emotional stakes and verification practices. Examining whether certain topics prolong confusion or intensify doubt could inform more targeted educational responses, such as health literacy or civic education initiatives.

Together, these directions point to the value of continued qualitative and mixed-method research that attends to context, development, and content. Such work can deepen understanding of how adolescents learn from encounters with misinformation and how educational support can be better aligned with their lived experiences.

## Conclusion

This study shows that adolescents are not simply passive recipients of misinformation. The relationships among initial acceptance, doubt, verification, recognition and reflective reassessment, and subsequent adjustment highlight adolescents’ capacity to reflect on error and develop more careful ways of engaging with digital information. These patterns were interrelated rather than fixed or universal, and participants differed in how they moved among them and in the forms of adjustment they described.

From an educational perspective, this process points to important moments for guidance and support. When educators and parents understand how adolescents experience confusion, questioning, and eventual recognition, they are better positioned to respond in ways that encourage learning rather than blame. Support offered at these moments can help adolescents move more quickly toward clearer judgment and more confident evaluation.

The findings also suggest that media literacy is not only a set of technical skills but a developmental process shaped by emotion, social interaction, and experience. Adolescents who work through misinformation episodes often emerge with stronger awareness of their own judgment and greater attention to credibility. Supporting this growth can contribute to a generation of young people who approach information with care, reflection, and a willingness to seek understanding. Such capacities are essential for sustaining trust and shared knowledge in contemporary information environments.

## Data Availability

The raw data supporting the conclusions of this article will be made available by the authors, without undue reservation.

## References

[ref1] Alderson P. Morrow V. (2020). The Ethics of Research with children and young People: A Practical Handbook. 2nd Edn. London: SAGE.

[ref2] Bandura A. (1986). Social Foundations of Thought and action: A social Cognitive Theory. Englewood Cliffs, NJ: Prentice-Hall.

[ref3] Bandura A. (1991). Social cognitive theory of self-regulation. Organ. Behav. Hum. Decis. Process. 50, 248–287. doi: 10.1016/0749-5978(91)90022-L

[ref4] Bandura A. (2009). “Social cognitive theory of mass communication,” in Media Effects: Advances in Theory and Research, eds. Bryant J. Oliver M. B.. 3rd ed (New York, NY: Routledge), 94–124.

[ref5] Beyens I. Frison E. Eggermont S. (2016). “I don’t want to miss a thing”: adolescents’ fear of missing out and its relationship to adolescents’ social needs, Facebook use, and Facebook-related stress. Comput. Hum. Behav. 64, 1–8. doi: 10.1016/j.chb.2016.05.083

[ref6] Braun V. Clarke V. (2006). Using thematic analysis in psychology. Qual. Res. Psychol. 3, 77–101. doi: 10.1191/1478088706qp063oa

[ref7] Braun V. Clarke V. (2021). One size fits all? What counts as quality practice in (reflexive) thematic analysis? Qual. Res. Psychol. 18, 328–352. doi: 10.1080/14780887.2020.1769238

[ref8] Breakstone J. Smith M. Wineburg S. Rapaport A. Carle J. Garland M. . (2021). Students’ civic online reasoning: a national portrait. Educ. Res. 50, 505–515. doi: 10.3102/0013189X211017495

[ref9] Corbelli G. Cicirelli P. G. D’Errico F. Paciello M. (2023). Preventing prejudice emerging from misleading news among adolescents: the role of implicit activation and regulatory self-efficacy in dealing with online misinformation. Soc. Sci. 12:470. doi: 10.3390/socsci12090470

[ref10] Gross E.-C. Balaban D. C. (2025). The effectiveness of an educational intervention on countering disinformation moderated by intellectual humility. Media Commun. 13:Article 9109. doi: 10.17645/mac.9109

[ref11] Halse M. Steinsbekk S. Bjørklund O. Hammar Å. Wichstrøm L. (2024). Emotions or cognitions first? Longitudinal relations between executive functions and emotion regulation in childhood. Child Dev. 95, 1508–1521. doi: 10.1111/cdev.14096, 38590290

[ref12] Hassoun A. Beacock I. Consolvo S. Goldberg B. Kelley P. G. Russell D. M. (2023) Practicing information sensibility: how Gen Z engages with online information Proceedings of the 2023 CHI Conference on Human Factors in Computing Systems 1–17 Association for Computing Machinery 10.1145/3544548.3581328

[ref13] Herrero-Diz P. Conde-Jiménez J. Reyes-de-Cózar S. (2020). Teens’ motivations to spread fake news on WhatsApp. Soc. Media Soc. 6, 1–14. doi: 10.1177/2056305120942879

[ref14] Herrero-Diz P. Conde-Jiménez J. Reyes-de-Cózar S. (2021). Spanish adolescents and fake news: level of awareness and credibility of information. Cult. Educ. 33, 1–27. doi: 10.1080/11356405.2020.1859739

[ref15] Jeong S.-H. Cho H. Hwang Y. (2012). Media literacy interventions: a meta-analytic review. J. Commun. 62, 454–472. doi: 10.1111/j.1460-2466.2012.01643.x, 22736807 PMC3377317

[ref16] Kolb D. A. (2014). Experiential Learning: Experience as the Source of Learning and Development. 2nd Edn. Upper Saddle River, NJ: Pearson Education.

[ref17] Lahti H. Kokkonen M. Hietajärvi L. Lyyra N. Paakkari L. (2024). Social media threats and health among adolescents: evidence from the health behaviour in school-aged children study. Child Adolesc. Psychiatry Ment. Health 18:Article 62. doi: 10.1186/s13034-024-00754-8, 38812043 PMC11138097

[ref18] Lazarus P. J. Costa A. (2020). “Teaching emotional self-regulation to children and adolescents,” in Fostering the Emotional well-Being of our Youth: A school-based Approach, eds. Wicks R. J. Maynard E. A. Lazarus P. J. (New York, NY: Oxford University Press), 264–281.

[ref19] Li W.-P. (2024). Truth Matters: The 2024 Survey on what Taiwanese Think about Misinformation and fact-Checking. New Taipei City, Taiwan: Taiwan FactCheck Center.

[ref20] Martel C. Pennycook G. Rand D. G. (2020). Reliance on emotion promotes belief in fake news. Cogn. Res. Princip. Implicat. 5:47. doi: 10.1186/s41235-020-00252-3, 33026546 PMC7539247

[ref21] McGrew S. (2020). Learning to evaluate: an intervention in civic online reasoning. Comput. Educ. 145:Article 103711. doi: 10.1016/j.compedu.2019.103711

[ref22] McGrew S. Breakstone J. Ortega T. Smith M. Wineburg S. (2018). Can students evaluate online sources? Learning from assessments of civic online reasoning. Theory Res. Soc. Educ. 46, 165–193. doi: 10.1080/00933104.2017.1416320

[ref23] Nickerson R. S. (1998). Confirmation bias: a ubiquitous phenomenon in many guises. Rev. Gen. Psychol. 2, 175–220. doi: 10.1037/1089-2680.2.2.175

[ref24] Notley T. Dezuanni M. (2019). Advancing children’s news media literacy: learning from the practices and experiences of young Australians. Media Cult. Soc. 41, 689–707. doi: 10.1177/0163443718813470

[ref25] Paciello M. Corbelli G. D’Errico F. (2023). The role of self-efficacy beliefs in dealing with misinformation among adolescents. Front. Psychol. 14:1155280. doi: 10.3389/fpsyg.2023.1155280, 37275715 PMC10233930

[ref26] Palinkas L. A. Horwitz S. M. Green C. A. Wisdom J. P. Duan N. Hoagwood K. (2015). Purposeful sampling for qualitative data collection and analysis in mixed method implementation research. Adm. Policy Ment. Health Ment. Health Serv. Res. 42, 533–544. doi: 10.1007/s10488-013-0528-y, 24193818 PMC4012002

[ref27] Pennycook G. McPhetres J. Zhang Y. Lu J. G. Rand D. G. (2020). Fighting COVID-19 misinformation on social media: experimental evidence for a scalable accuracy-nudge intervention. Psychol. Sci. 31, 770–780. doi: 10.1177/0956797620939054, 32603243 PMC7366427

[ref28] Qamaria R. S. Kuswandi D. Setiyowati N. Bahodirovna A. M. (2025). Digital resilience in adolescence: a systematic review of models, methods and theoretical perspectives. Multidiscip. Rev. 8:2025287. doi: 10.31893/multirev.2025287

[ref29] Tandon A. Dhir A. Almugren I. AlNemer G. N. Mäntymäki M. (2021). Fear of missing out (FoMO) among social media users: a systematic literature review, synthesis and framework for future research. Internet Res. 31, 782–821. doi: 10.1108/INTR-11-2019-0455

[ref30] Tracy S. J. (2024). Qualitative Research Methods: Collecting Evidence, Crafting Analysis, Communicating impact. 3rd Edn. Hoboken, NJ: Wiley.

[ref31] Vraga E. K. Bode L. (2020). Defining misinformation and understanding its bounded nature: using expertise and evidence for describing misinformation. Polit. Commun. 37, 136–144. doi: 10.1080/10584609.2020.1716500

[ref32] Wineburg S. McGrew S. (2016). Evaluating Information: The Cornerstone of civic Online Reasoning. Stanford, CA: Stanford History Education Group.

[ref33] Wineburg S. McGrew S. (2019). Lateral reading and the nature of expertise: reading less and learning more when evaluating digital information. Teach. Coll. Rec. 121, 1–40. doi: 10.1177/016146811912101102

